# Glycated Hemoglobin (HbA1c) as a Biomarker for Diabetic Foot Peripheral Neuropathy

**DOI:** 10.3390/diseases9010016

**Published:** 2021-02-22

**Authors:** Giulia Casadei, Marta Filippini, Lorenzo Brognara

**Affiliations:** 1 Medical Clinic of Doctor Accorsi, Via della Ghisiliera 5, 40123 Bologna, Italy; giuly.casadei@gmail.com (G.C.); marta.filippini1@gmail.com (M.F.); 2 Department of Biomedical and Neuromotor Science, University of Bologna, Via Ugo Foscolo 7, 40123 Bologna, Italy

**Keywords:** HbA1c, diabetic peripheral neuropathy, neuropathy, diabetes, diabetic foot complications

## Abstract

Background: Diabetic peripheral neuropathy (DPN) is known to predict foot ulceration, lower-extremity amputation and mortality. Patients with diabetes mellitus have a predisposition toward developing chronic inflammatory demyelinating polyneuropathy, and this may also facilitate the formation of diabetic foot and cutaneous impairment, which are considered one of the most serious impairments of diabetes mellitus, with a prevalence of 4–10% in this population. Biomarkers research provides opportunities for the early diagnosis of these complications for specific treatments useful to prevent amputation and, therefore, physical inability and mental disturbance. The recent literature has suggested that glycemic levels may be a novel factor in the pathogenesis of diabetic foot complications and is an important mediator of axonal dysfunction. The aim of this systematic literary review is to determine whether hemoglobin A1c (HbA1c) is a positive predictor for diabetic foot peripheral neuropathy and its complications, such as foot cutaneous impairments. There is a lack of consensus regarding the effect of glycemic variability on diabetic foot peripheral neuropathy, unlike other complications such as retinopathy, nephropathy or micro/macrovascular pathology. Methods: Relevant articles were searched in the Medline database using PubMed and Scopus and relevant keywords. The primary search terms used were “glycated hemoglobin” OR “HbA1c” AND “diabetic neuropathies” AND “Foot”. Results: A number of articles (336) were initially identified while searching the scientific literature regarding this topic, and 32 articles were selected and included in this review. Conclusions: This review highlights the role of HbA1c in diabetic foot peripheral neuropathy. Biomarkers play an important role in the decision-making process, and HbA1c levels are extensively used for diabetic foot clinical outcomes and settings, but biomarker research in diabetic foot peripheral neuropathy is in its infancy and will require careful attention to a number of factors and associations, since the consequences of DPN also include neurological alterations. HbA1c is an accurate and easy-to-administer test and can be an effective biomarker in establishing the diagnosis of diabetes, but future research should focus on standardizing the HbA1c level and selecting which DPN value and its correlated complications, such as foot cutaneous impairments, are the most informative.

## 1. Introduction

Diabetes mellitus (DM) is considered to be a serious public health problem due to its high prevalence and related complications, among which is diabetic peripheral neuropathy (DPN). DPN is a disease often associated with neuropathic pain, foot ulceration and lower extremity amputation, which can significantly affect the quality of life of patients [1,2]. The most frequent type of neuropathy associated with diabetic foot complications is the distal symmetric sensorimotor polyneuropathy, and, along with peripheric vascular disease, it is a major contributing factor to the formation of foot ulcers. The control of the disease relies both on individual actions for self-care and on medical treatments and surveillance. A healthy, intact diabetic foot is indeed best maintained by a consistent and recurrent preventive treatment strategy accomplished through a multidisciplinary approach that encompasses instruction in glucose assessment, insulin and other diabetes medication administration; diet; daily foot inspection and care; proper footwear and the necessity for prompt treatment of new lesions. Regarding medical surveillance, a common strategy to evaluate the effectiveness of DM treatment is the use of a biomarker. By definition, a biomarker is a “characteristic that is objectively measured and evaluated as an indicator of normal biological processes, pathogenic processes or pharmacological responses to a therapeutic intervention” [3]. Specifically, for the case of DM, the levels of glycated hemoglobin (HbA1c or hemoglobin A1c) are periodically measured, as glycemic variability has been recognized as the most important risk factor for DPN.

Early detection and good glycemic control be proven to prevent adverse outcomes associated with DPN, but there is a lack of consensus regarding the effect of glycemic variability on diabetic foot peripheral neuropathy alterations such as cutaneous impairment [4,5,6]. Various etiologies have been suggested to describe the pathogenesis of diabetic neuropathy and its relationship with hyperglycemia [7,8,9,10]. One example was provided, among others, by the Banting Memorial Lecture (2004), mentioning how increased cytosolic glucose causes an accelerated transformation of glucose to sorbitol by aldose reductase with the consumption of free cytosolic NADPH and production of NADP+ [11].

HbA1c provides the better measure, as it reflects levels of blood glucose over several weeks, and it is the main method of monitoring glycaemia in diabetes; for this reason, the purpose of our study was to review articles published from 2010 to 2020 in order to analyze the relation between diabetic foot peripheral neuropathy-related and HbA1c genetic markers in the literature.

### 1.1. Diabetic Peripheral Neuropathy

Patients with diabetes occasionally develop diabetic polyneuropathy, which is characterized by both positive symptoms such as pain and negative symptoms such as numbness/dysesthesia [12]. Diabetic peripheral neuropathy, defined as “the presence of symptoms and/or signs of peripheral nerve dysfunction in people with diabetes after the exclusion of other causes” is the leading cause of polyneuropathy, affecting up to 50% of older type 2 diabetic patients [13,14,15,16,17,18]. In the management of this condition, treatment centers on control of the patient’s blood sugar level as the first step. Chronic and acute sensory neuropathy are usually precipitated by an episode of glycemic instability (such as ketoacidosis), often accompanied by autonomic dysfunction with late sequelae, which includes foot ulcerations [19]. In addition, achieving stable blood glucose control is also extremely important in terms of painful symptoms management [20].

Sensory symptoms associated with the disease are extremely variable and can be divided into positive—burning, tingling, sharp, dull and/or searing feeling—and negative symptoms—numbness, dysesthesia, loss of balance, heaviness in the legs, stiffness or feelings of something bunched up on the ball or sulcus of the foot [12,21].

Neuropathic symptoms are equally variable and include spontaneous sensations (paresthesia), unpleasant sensations (dysesthesias) or hypersensitivity (hyperalgesia) to pressure or touch but, also, numbness, tingling, unsteadiness, prickling or burning pain in the legs and/or feet. Neuropathic signs were defined as reduced or absent ankle reflexes (using an appropriate reflex hammer) and reduced or absent distal sensation, including a vibration perception (using a 128-Hz tuning fork), touch sensation (using a 10-g monofilament), thermal discrimination (using cold and warm objects), pinprick sensation (using a pin) and proprioception. Patients with DPN also suffer from an altered gait strategy and present a fivefold increased risk of falling [22,23,24,25,26,27]. Moreover, DPN causes sleep interferences, mood disorders and, more in general, a poor health-related quality of life [28]. The progression of DPN leads to a loss of the protective sensation, skin ulcerations and chronic wounds. A lack of awareness and inappropriate management of DPN has led to much unnecessary lower limb amputations, despite the fact that the importance of DPN in the etiopathogenesis of foot ulcerations has been confirmed by numerous studies [29].

Neuropathy is assessed by a variety of techniques: signs are determined using a modified neuropathy disability score (NDS) derived from abnormalities of pain sensation using a Neurotip™, Achilles reflex using a tendon hammer, vibration sensation using a 128-Hz tuning fork and dorsal temperature sensation using warm and cool rods. Cutaneous perception can be detected with a simple neurologic examination of the lower extremities involving the use of a 10-g Semmes Weinstein monofilament, to test sensation, or a composite score such as a modified neuropathy disability score [30]. The Michigan Neuropathy Screening Instrument (MNSI) is a simple, sensitive and specific tool for the screening of DPN validated in many cohort and clinical trial studies [31,32,33].

Thus, all patients with a neuropathic deficit must be considered as being at risk of foot ulceration and require regular podiatric assessments.

Various options are then used to treat the painful symptoms.

A large number of relationships exist between a new diabetic foot ulcer and its potential predictors; some have the strongest evidence, such as a history of foot ulcers or history of amputation, and others will have to be investigated [34].

### 1.2. Cutaneous Foot Impairment

The frequency of cutaneous impairment in diabetic patients has been reported to range from 30.0% to 91.2%, but its pathogenesis has yet to be elucidated [35,36,37]. Although diabetic foot cutaneous symptoms may not be life-threatening, they may seriously affect the quality of life and serve as external markers for extracutaneous complications, which are strongly associated with DPN [38,39,40]. Carrington et al. (2002) observed how the motor nerve conduction velocity, frequently assessed in clinical trials of diabetic peripheral neuropathy, can predict foot ulcerations [41].

On the other hand, hyperglycemia affects keratinocytes and fibroblast activities, and the combination with diabetic neuropathy may play an important role in the pathogenesis of diabetic foot complications and amputation [42,43].

These two aspects are closely related, as the pathogenesis initially involves unrecognized trauma within skin areas of neuropathy, while hyperglycemia affects chemotaxis, resulting in a badly disturbed cell proliferation and migration healing process [42].

**Figure 1:**
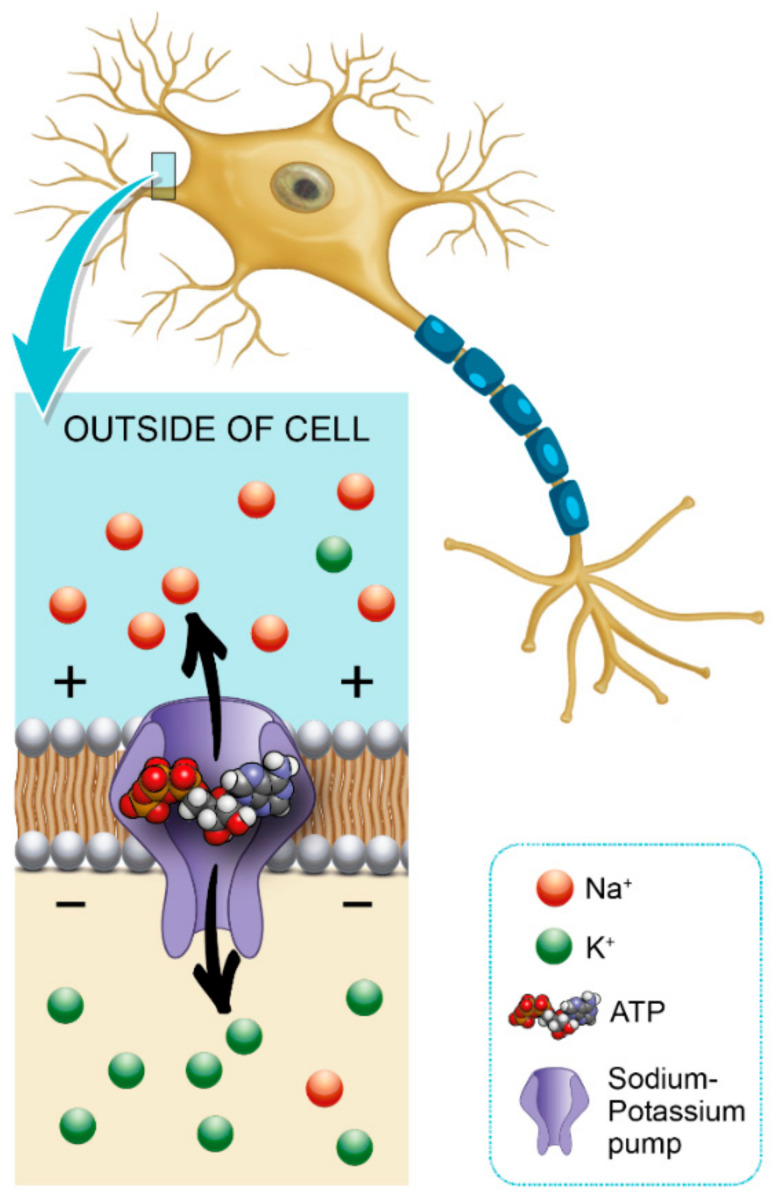
Role of hemoglobin A1c (HbA1c) in Na^+^/K^+^ pump dysfunction and diabetic peripheral neuropathy (DPN) pathogenesis.

Glycemic variability is an important factor that contributes to axonal ion channel dysfunction, a key mediator in axonal degeneration in diabetes mellitus type 1 [44]. Patients with diabetes mellitus have a predisposition to develop chronic inflammatory demyelinating polyneuropathy, and this may also facilitate the formation of a diabetic foot [45]. Hyperglycemia leads to a shunting of excessive glucose through an activated polyol pathway that disrupts neural Na^+^ /K^+^ -ATPase, causing intra-axonal Na^+^ accumulation (Figure 1). This pattern of change is consistent with axonal depolarization, an abnormality that may occur in the context of dysfunction of the energy-dependent axonal Na^+^/K^+^ pump [46].

Increased glucose may also affect the skin, a phenomenon that occurs in about 30% of people with diabetes that may be a precursor of the disease [47,48]. Hemoglobin A1c (HbA1c) reflects glycemia over two to three months, and according to the guidelines set forth by the American Diabetes Association, the goal of type 2 diabetes therapy is to reduce glycated hemoglobin A1c (HbA1c) to 7% or 6.5% [49].

Elevated HbA1c levels would mostly be associated with poor wound healing, and HbA1c is a good biomarker for foot ulcer outcomes (wound healing time) in diabetic patients [50].

## 2. Methods

### 2.1. Search Strategy and Selection Criteria

**Figure 2:**
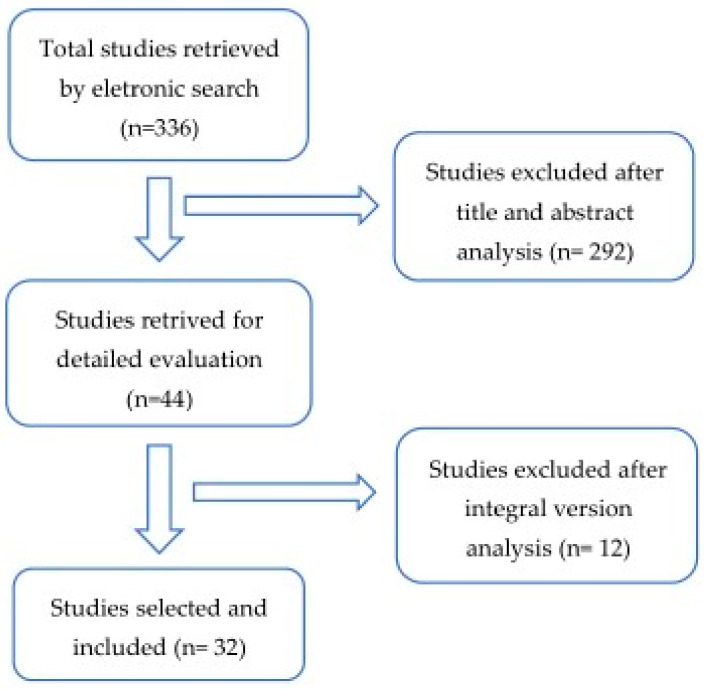
Flow diagram of the articles selection process through a systematic review.

Databases and Literature Search: An electronic search was performed in PubMed for all relevant literature published up to 25 November 2020. The search terms were the following: (“glycated hemoglobin a” (MeSH Terms) OR “glycated hemoglobin a” (All Fields) OR “hba1c” (All Fields) OR “hba1cs” (All Fields)) AND (“diabetic neuropathies” (MeSH Terms) OR (“diabetic” (All Fields) AND “neuropathies” (All Fields)) OR “diabetic neuropathies” (All Fields) OR (“neuropathy” (All Fields) AND “diabetic” (All Fields)) OR “neuropathy diabetic” (All Fields)) AND (“foot” (MeSH Terms) OR “foot” (All Fields)). We supplemented our search by manually reviewing the references of all eligible studies (Figure 2).

### 2.2. Eligibility Criteria

The following inclusion criteria were fulfilled: (a) observational studies (cross-sectional, case–control or cohort study) that allowed for the assessment of a causal association between HbA1c, DPN and diabetic foot complications; (b) the definition of DPN given in the studies included only sensory neuropathy, not Charcot neuroarthropathy and (c) the studies compared HbA1c levels between groups with and without DPN and diabetic foot complications. We excluded conference proceedings and articles reporting results from less than 10 patients and that did not assess diabetic foot peripheral neuropathy complications related to HbA1c values.

## 3. Results

### 3.1. Characteristics of Included Studies

We identified 32 studies that met our inclusion criteria for a systematic review (Table 1 and Table 2). Two-thirds of the studies included were prospective or retrospective cross-sectional cohort studies, and all of the 32 studies investigated the relationship between HbA1c and DPN. Type 2 diabetes was assessed in 25 studies, type 1 in four studies and type 1 and type 2 in four studies.

Twenty-six studies involved more than 100 participants, but only five studies included healthy adults as a control group.

Table 1 and Table 2 summarize the characteristics of the 32 identified studies.

### 3.2. Protocols and Characteristics of Studies Examining the Interacting Mechanisms between HbA1c Levels and Diabetic Foot Peripheral Neuropathy

Three studies recorded no significant effect of elevated HbA1c levels or intensive glycaemia therapy on the peripheral neuropathy [51,59,62].

Thirty studies observed that an increase in HbA1c variability is closely associated with DPN in diabetic patients.

Regarding DPN, the clinical features in diabetic patients enrolled, in the majority of studies, were classified using the MNSI staging scale, but, in this review, we did not find homogeneity, since a wide variety of tests and instruments such as electrodiagnostic techniques or nerve conduction studies were reported.

The two studies that tested the effects of HbA1c on diabetic foot peripheral neuropathy showed that the strong relationship between HbA1c values and vibration perception threshold (VPT) could be a predictor for complications in the foot following DPN [56,57].

Kamran M. Hassan et al. in 2016 observed a strong association between HbA1C and neuropathy, leading to a high risk of diabetic foot, since poor footwear, neuropathic foot and ulceration and higher HbA1c levels were interlinked in terms of the pathogenesis (footwear can play a critical role in the pathogenesis of foot complications in diabetic patients with DPN).

In the clinical research community, a consensus on the relationship between hyperglycemic and diabetic foot complications in patients with DPN is still to be reached; this is also true in terms of the cut-off point of HbA1c needed to predict DPN. There is a lack of studies analyzing various items of DPN in light of the foot complications, which would have been interesting in order to statistically compare various impairments and understand which HbA1c values can be predictive and associated with diabetic foot complications. Some of these complications can be investigated in terms of the increased plantar pressures, as done by Mohammed R. Halawa in 2017 [56], and associated foot deformities and risk factors such as ulcers, calluses, dry skin, deformities, footwear condition, dry skin, bunions, fissures, callus and ingrown nails, many of which are already present in the MNSI questionnaire.

The positive effects of intensive glycaemia therapy may add an important benefit to reduce the risk of ulcers, with fewer diabetes-related foot complications.

## 4. Discussion

Biomarkers play a key role in the diagnosis, prognosis and clinical management of various chronic diseases.

Despite these efforts, we were unable to find any clinical trials successfully investigating the impact of glycemic control and foot complications correlated to DPN. Diabetic foot peripheral neuropathy often cooccurs with other diabetes-induced complications. Different aspects related to diabetic foot syndrome, such as gait alterations, psychological complaints and even disorders, can affect the quality of life of these patients; moreover, there is a general gender-dependent higher prevalence of diabetic foot impairment in men, although this was shown to be dependent on the geographical area [83,84].

We evaluated several foot alterations consisting of the most frequently observed in a diabetic foot according to the Michigan Neuropathy Screening Instrument, but, in this review, we did not find one particular glycated level used to predict diabetic foot peripheral neuropathy alterations. A consensus in this regard in the clinical research community is still to be gained in terms of foot cutaneous impairment or other foot complications associated with DPN and a high level of HbA1c.

The measurement of high levels of HbA1c could be a strategic biomarker to detect diabetic foot peripheral neuropathy. Indeed, intensive glycemic control and lower levels of HbA1c are followed by a reduction in diabetic complications: in HbA1c, <7% is associated a 60% reduction in the incidence of peripheral neuropathy [85].

The use of HbA1c level as an indicator of the severity of polyneuropathy and poor glycemic control (HbA1c level >6.5%) could significantly increase the risk and quantitatively reflect the severity of polyneuropathy in diabetic patients [82]. Evidence suggests that a high level of HbA1c can lead to diabetic peripheral neuropathy, so patients with high levels of HbA1c should be considered to be at the potential risk of diabetic foot complications—foot ulcerations or injuries—that frequently occur in DPN and should receive preventive education from a podiatrist. However, although the majority of studies observed that an increase HbA1c variability is closely associated with DPN in diabetic patients, in contrast, Laura Mayeda, Piotr Dziemidok and Faramarz Ismail-Beigi conversely recorded no significant effects of elevated HbA1c levels or intensive glycemia therapy on peripheral neuropathy. Besides these contrasting results, further studies should investigate the relationship between hyperglycemic and DPN, possibly including a comparison of the different instruments used to assess the clinical features. The crucial issue of defining a relationship between a glycemic control and DPN impairments has not been possible due to the critical bias listed above. Only eight (Table 1) out of 32 studies analyzed the presence of diabetic foot peripheral neuropathy and related problems such as diabetic foot and cutaneous impairment; this lack of data makes a meta-analysis or a statistical investigation impossible to carry out in order to find an association between HbA1c and DPN.

## 5. Conclusions

The purpose of this literature review was to evaluate the common and interacting mechanisms between HbA1c levels and diabetic foot peripheral neuropathy.

According to high-quality evidence, enhanced glucose control significantly prevents the development of clinical neuropathy [86]. The exact role that intensive glycemic control has in treating diabetic foot complications in patients with diabetic peripheral neuropathy requires further investigation. All patients with diabetes and sensory loss require regular podiatric care and should have a thorough foot examination. The significant reductions in the development of peripheral neuropathy, if further sustained, suggest that intensive glycemia therapy could decrease the risk of ulcers and the number of future leg amputations, reducing diabetes-related foot complications and, thus, significantly improving the quality of life of patients.

Many dermatological foot complications are caused by hyperglycemia, and the pathogenesis is also caused by neuropathy. Improved glycemic control has been shown to have a sustained benefit on diabetes and its complications, but evidence of the effects of HbA1c as a biomarker on some of the diabetic foot peripheral neuropathy complications such as hyperkeratosis, pre-ulcerative lesions or xerosis is still lacking.

## Figures and Tables

**Table 1:** Studies examining the usefulness of hemoglobin A1c (HbA1c) as a biomarker for the early detection of diabetic peripheral neuropathy (DPN) and foot impairment. In this table, we excluded studies that did not report HbA1c values or did not assess the prevalence of a diabetic foot.

Reference	Type of Study	Participants (Number and Age)	Male/Female	Type of Diabetes	HbA1c	DPN and Assessment Tool	Diabetic Foot and Cutaneous Impairment	Conclusion
Piotr Dziemidok (2012) [51]	Observational study	204 patients mean age 59.2 ± 11.7; DPN T1D = 14; DPN T2D = 113	117 M 87 F	type 1 N = 29 type 2 N = 175	The mean glycated hemoglobin level was assessed in 8.53% ± 1.87%	MNSI, monofilament (Semmes-Weinstein 5.07–10 g), a 128-Hz calibrated tune-fork for the vibration perception test and Tip-Therm to assess temperature sensation	N = 61 decreased sensation of touch; N = 120 decreased sensation of temperature; N = 61 decreased sensation of vibration; N = 21 foot ulcers; N = 9 Charcot arthropathy	There was no correlation between the prevalence and severity of peripheral, sensorial neuropathy and current diabetes control evaluated as the level of HbA1c in patients with long-term established diabetes.
Yih-Kuen Jan (2018) [52]	Clinical experimental study	26 patients 18 DM + DPN age: 48.5 ± 9.4 8 HC aged 21.8 ± 2.4	17 M 9 F	Type 2	HbA1c: 7.8 ± 0.9%	DPN was confirmed by the inability to sense the location and pressure applied by a 5.07 Semmes-Weinstein monofilament	Skin blood flow between the plantar and dorsal side of the foot	People with DM and peripheral neuropathy had a higher skin blood flow on the plantar foot compared to the dorsal foot, and the increased blood flow was attributed to higher metabolic and lower myogenic controls.
Hala O. El-Mesallamy (2011) [53]	Cohort study	80 patients. HC (n = 20, age 55 ± 3.1 years). T2DM with no DPN (n = 30). T2DM with DF and DPN (n = 30, age 55 ± 3.9 years).	80 M	Type 2	Healthy: 5 ± 0.56% DM with No DF no DPN: 9.65 ± 0.94% DM with DF and DPN: 10.8 ± 0.76%	Vibratory perception threshold/temperature discrimination, tests of pinprick sensation, ankle reflex assessment and monofilament examination	A DF case was determined based on the “Wagner classification”	This study highlighted the opportunities for further research that would definitively establish the AGE-RAGE axis as important in investigations of diabetic foot and, also, in terms of an endogenous protection factor against the occurrence of DF
Rainha J. de Souza (2015) [54]	cohort study	153 patients (51 HG, 50 with presymptomatic DPN and 52 with symptomatic DPN (47.4 mean age)	76 M 77 F	Type 2	≤6% HC, Presymptomatic diabetics 8.1 (range 6–14); Symptomatic diabetics 9.2 (range 6–13)	Electrodiagnostic techniques were employed to evaluate right-sided peroneal motor, median and ulnar motor and sensory and sural sensory responses	47.1% reported sensory loss in the feet, and mild unsteadiness of gait was noted in 53.8%.	Nerve conduction changes in DPN follow a predictable pattern, correlating with clinical features and long-term glycemic control. HbA1c levels were related to prolonged sensory latencies in the upper limbs but not the lower.
Laura Salvotelli (2015) [55]	Retrospective study with cross-sectional analyses	3591 patients 66.59 ± 10.09 years (NO DPN) 72.24 ± 9.46 year (with DPN)	1998 M (55%), 1593 F (44.5%)	Type 2	HbA1c (%) no DPN: 7.34 ± 1.35. DPN 7.54 ± 1.51	MNSI questionnaire, monofilament, VPT and grading of ankle reflexes	Foot inspection for the presence of deformities	Type 2 diabetic patients with a negative medical history of diabetic foot problems had 30% probable somatic neuropathy. Age (or disease duration), HbA1c, BMI and ABI were significant predictors of neuropathy.
Arun G. Maiya (2019) [56]	Cross-sectional study	534 patients (mean age 56.8 ± 12.8)	Not reported	Type 2	HbA1c was categorized 8.7% ± 2.1% into 2 groups: >6.0% and <6.0%	Neurotouch beta version (monofilament, VPT probe, infrared thermometry and hot and cold perceptions)	Dry skin, bunions, fissures, calluses, ingrown nails and hammer toes	There is strong relationship between HbA1c values and the vibration perception threshold (VPT) and could be a predictor for complications in the foot following DPN.
Mohammed R. Halawa (2017) [57]	Cross-sectional study	80 patients aged between 18 and 60 years Group I: 20 with neuropathy Group II: 30 patients without neuropathy. Group III: 30 control healthy volunteers.	29M (18%) 51F (81%)	Type 2	8.40 ± 1.12 for patients with a <10-y diabetes duration 10.57 ± 1.52 for patients with a >10-y diabetes duration	10-g monofilament or vibration, touch, pain, ankle reflex, inspection for the presence of any foot deformity and, finally, by undergoing the Douleur Neuropathique 4 (DN4) questionnaire score.	Plantar pressure was recorded for all patients using the Mat-scan (Tekscan, Inc., ver. 6.34, Boston, MA, USA) in static and dynamic conditions. Various foot deformities were also assessed.	Persons with diabetic neuropathy have elevated peak plantar pressure compared to patients without neuropathy and the control group. HbA1c % as a surrogate for glycemic control had no direct impact on peak planter pressure, yet it indirectly impacts neuropathy evolution throughout the disease duration, eventually leading to drastic planter pressure and gait biomechanics changes.
Kamran M Hassan (2016) [58]	cross sectional analytical study	222 patients (55.4 ± 11.1 years) T1D = 33 T2D = 189	145 M (65.3%) 77 F (34.6%)	Type 1 and 2	HbA1c was 8.6% ± 2.1%	Risk classification system of IWGDF: pinprick, temperature, vibration perception (using a 128- Hz tuning fork), 10-g monofilament pressure sensation at the distal halluces and ankle reflexes	Feet risk factors: ulcers, calluses, dry skin, deformities and footwear conditions	The prevalence of neuropathy was seen in 62 (27.93%) patients. A number of patients (17.7%) were illiterate, and their footwear or shoes were inappropriate. The poor footwear, neuropathic foot and ulceration and higher HbA1c levels are interlinked in terms of pathogenesis developing again and again.

HC: healthy control subjects, DPN: diabetic peripheral neuropathy, DM: diabetes mellitus, T1D and T2D: type 1/2 diabetes, MNSI: Michigan Neuropathy Screening Instrument, DF: diabetic foot, BMI: body mass index and AGE: advanced glycation end products.

**Table 2:** Studies examining the usefulness of HbA1c as a biomarker for the early detection of DPN.

Reference	Type of Study	Participants (Number)	Participants (Mean Age)	Male/Female	Type of Diabetes	HbA1c	DPN Value and Assessment Tool	Conclusion
Biserka Kovač (2011) [59]	Clinical observational study	100	61	41 M 59F	Type 2	from <6.5% to >7.0%	Crude muscular strength, touch sensation and vibration (with tuning fork). All patients underwent detection electromyography, neurographic analysis and Neuropathy Total Symptom Score (NTSS-6)	The lower or absent sense of vibration correlated significantly with the higher values of HbA1c in 88% of the patients with poorly regulated glycemia. Higher values of glycemia determined by HbA1c are a significant predictor of electrophysiological changes of peripheral nerves in the case of diabetic polyneuropathy.
Mohsin Azam (2015) [60]	Prospective cohort study	1488	55.75 ± 11.02	820 M 668 F	Type 2	48 mmol/mol (6.5%)	vibration perception threshold > 25 volts measured by a neurothesiometer	HbA1c < 48 mmol/mol may not exclude clinically important diabetes; in fact, at recruitment and one year, there were no between-group differences in the prevalence of diabetic complications, except that those diagnosed with HbA1c < 48 mmol/mol had more sensory neuropathy at recruitment.
MdlA Lazo (2014) [61]	Cross-Sectional Study	129	59.2	56 M 73 F	Type 2	MeanHbA1c was 8.7%	DNS score and Semmes-Weinstein monofilament test	More than half of the T2DM patients had peripheral neuropathy when evaluated by the DNS score and SWF test. Associated factors to DPN included being a diabetic patient for over ten years and receiving insulin plus metformin.
Faramarz Ismail-Beigi (2010) [62]	Randomized trial	10,251	62	Not reported	Type 2	≥7.5%	MNSI, 128-Hz tuning fork and 10-g force monofilament test	The study recorded no significant effect of intensive glycaemia therapy on the peripheral neuropathy.
Hande Türkyilmaz (2017) [63]	Retrospective study	111	138 months	59 M 52 F	Type 1	>7.51 and ≤9	nerve conduction test	Poor metabolic control, especially during early stages of the disease, is a major risk factor for neuropathy development.
Y. Unmar (2017) [64]	Cross-sectional study	240	56.3 ± 11	156 M 84 F	Type 2	9.29 ± 2.3%	DPN was assessed using pinprick, using a 128-Hz tuning fork, using the 10-g monofilament and Nicolet Viking Quest EMG machine	Patients with DPN tended to be older, have a longer duration of the disease, elevated HbA1c levels, a higher prevalence of atherosclerotic changes in the carotid and/or lower limb vessels, diabetic retinopathy, diabetic nephropathy and hypertension compared to patients without DPN.
Laura Mayeda (2019) [65]	Prospective observational cohort study	105	68	67 M 38 F	Type 2	7.8%	Michigan Neuropathy Screening Instrument. DPN = 74%	Lower time-in-range glucose was associated with DPN symptoms. Laboratory values of HbA1c were not found to be associated with DPN.
Jian‑bin Su (2018) [66]	Cross-sectional observational study	563	56.4 ± 9.8	299 M 264 F	Type 2	M-HbA1c (%) 8.85 ± 1.20	Electromyogram, reflex hammer, a 128-Hz tuning fork, 10-g monofilament and pin for pinprick sensation.	Increased HbA1c variability is closely associated with DPN in type 2 diabetic patients and could be considered as a potent indicator for DPN in these patients.
Yun-Ru Lai (2019) [67]	Observational study	223	62.7 ± 9.6	145 M 78 F	Type 2	HbA1c (%) 7.5 ± 1.0	The nerve conduction was performed using Nicolet Viking machines.	HbA1c variability plus chronic glycemic impairment is strongly associated with the severity of peripheral neuropathy in patients with type 2 diabetes.
Fukashi Ishibashi (2019) [68]	Cohort study	158	50.4	93 M 65 F	Type 2	HbA1c 9.6%	Vibration perception, pinprick, temperature perception and ankle reflexes.	The normalized HbA1c levels are more effective than standard care for preventing the development of neuropathy.
Alon Abraham (2017) [69]	Cross-sectional cohort study	164	55.75	26 M 27 F 84 M/79 F (T2D)	Type 2	6.0–6.4% to 6.5–7.4%	Nerve conduction studies (NCS) and vibration perception thresholds (VPT) using a neurothesiometer	Early signs of subclinical small nerve function impairment are seen in healthy controls at HbA1c levels of 5.5–6%. The most prominent decline in both small and large nerve fiber functions was seen with less impaired glycemic control and a shorter duration of diabetes at HbA1c levels of 6.5–7.4%, compared with >7.5%.
Paul D. Loprinzi (2013) [70]	Cross sectional study	339	61.8	178 M 161 F	Type 2	≥6.5%	Standard monofilament (5.07 Semmes-Weinstein nylon monofilament)	Modest levels of moderate-to-vigorous intensity physical activity coupled with glycemic control may help prevent or slow the progression of diabetic end-organ damage, particularly diabetic neuropathy.
Dong D. Wang (2014) [71]	Cross-Sectional Study	154	52	346 M 206 F	Type 1 and 2	9.0%	Neurothesiometer,10-g Semmes-Weinstein monofilament and disposable pin. The observed prevalence of DPN was 19.9%.	Diabetes duration and glycemic control were strongly associated with DPN.
Li Li (2014) [72]	Cross-sectional study	3359	62.3 ± 11.2	1607 M 1752 F	Type 2	8.75 ± 2.19%	10-g Semmes-Weinstein monofilament at the hallux of each foot. Prevalence of DPN was 33.1% (1113 patients).	DPN is prevalent in Chinese patients with T2DM who are overweight or obese. Age, HbA1c and duration of DM are associated with the presence of DPN.
Mamta Jaiswal (2017) [73]	Prospective cohort study	1992	22	863 M 872 F 175 M 83 F (T2D)	Types 1 and 2	9.1 ± 1.8%	Michigan Neuropathy Screening Instrument. The prevalence of DPN was 7% in youths with T1D and 22% in youths with T2D.	Interventions in youths that address poor glycemic control and dyslipidemia may prevent or delay debilitating neuropathic complications.
M. Peterson (2017) [74]	10-year follow-up study	87	71.1	46 M 41 F	Type 2	7.5 %	Nerve conduction studies	The HbA1c level was found to be associated with the amplitude of the sural nerve. Early detection is therefore likely to be important for the prevention of neuropathy in people with impaired glucose tolerance and type 2 diabetes.
Chun-Pai Yang (2015) [75]	Retrospective cohort study	37,375	60	18,331 M 19,044 F	Type 2	From 6% to 10%	DPN was determined through record linkage with ambulatory and in-patient care data in the National Health Insurance Research database.	Patients with type 2 DM and HbA1c ≥7.0% exhibit an increased risk of DPN, and the incidence of DPN is also associated with poor glucose control and cardiovascular risk factors.
Barbara H. Braffett (2020) [76]	Cohort Study	1441	27	52% M 48% F	Type 1	8.80%	DPN was assessed by a neurologist defined by symptoms, signs and nerve conduction study abnormalities in 2 or more nerves.	In summary, in these comprehensive analyses, the authors found that a higher mean HbA1c and older age were the strongest risk factors for both DPN and CAN.
Adams (2019) [77]	Cross-sectional study	236	58.6	78 M 158 F	Type 2	7.1%	DPN tested by 10-g monofilament at four plantar sites per foot and a 28-Hz tuning fork and neurothesiometer at the hallux.	Over a third of people with previously diagnosed diabetes had evidence of peripheral neuropathy with a loss of protective sensation.
Feng Xu (2014) [78]	Observational Study	90 patients	59.8 ± 8.3	46 M 44 F	Type 2	6.4 ± 0.4	10-g monofilament on four sites per foot, tendon hammer and 128-Hz tuning fork	There was a close relationship between glycemic variability evaluated by MAGE and DPN in type 2 diabetes with well-controlled HbA1c. DPN patients with well-controlled HbA1c showed a higher glycemic variability.
Christopher H. Gibbons (2017) [79]	Long term follow-up study	26 patients	35.4 ± 4.0	4 M 22 F	Type 1	7.6 ± 1.1 %	NIS-LL tool: A 88-point system that grades neuropathy from 0 (no neuropathy) to 88 (total loss of sensation, reflexes and strength in the legs). Likert scale for pain.	19/26 with stable glycemic control had improvement in neuropathy, pain and microvascular complications, while the 7/26 with unstable glycemic control had significant worsening of neuropathy, pain and microvascular complications.
Yen-Wei Pai (2018) [80]	Case–control, retrospective study	626	72.9 ± 10.5	333 M 293 F	Type 2	7.4 ± 1.4%	Michigan Neuropathy Screening and Douleur Neuropathique 4 (DN4) questionnaire	Long-term variability as evaluated by FPG-CV was associated to the risk of painful diabetic peripheral neuropathy in adults with T2D.
Sheyu Li (2019) [81]	Retrospective cohort study	21,352	63.3 ± 11.1	11,664 M 9688 F	Type 2	7.7 ± 2.0 %	13,111 (61.4%) patients. Value and assessment tools: not reported.	When considering the HVS, the clinicians can review the HbA1c profile for an individual; those where >60% of the measures vary by >0.5% are at high risk. A higher HbA1c variability is associated with increased risks of all-cause mortality, cardiovascular events and microvascular complications of diabetes independently of high HbA1c.
Won-Jae Lee (2016) [82]	Retrospective analysis	187	NR	128 M 59 F	Type 2	8.14 ± 1.63 %	Electrodiagnostic testing including NCS were performed by rehabilitation physicians using a Keypoint EMG machine	An increased HbA1c level is indicative of a state of chronic hyperglycemia and is a risk factor for polyneuropathy in diabetic patients and a quantitative measure of its severity.
